# Weight Gain following Pallidal Deep Brain Stimulation: A PET Study

**DOI:** 10.1371/journal.pone.0153438

**Published:** 2016-04-12

**Authors:** Paul Sauleau, Sophie Drapier, Joan Duprez, Jean-François Houvenaghel, Thibaut Dondaine, Claire Haegelen, Dominique Drapier, Pierre Jannin, Gabriel Robert, Florence Le Jeune, Marc Vérin

**Affiliations:** 1 “Behavior and Basal Ganglia” research unit (EA 4712), University of Rennes 1, Avenue Léon Bernard, Rennes, France; 2 Department of Neurophysiology, Rennes University Hospital, rue Henri Le Guilloux, Rennes, France; 3 Department of Neurology, Rennes University Hospital, rue Henri Le Guilloux, Rennes, France; 4 Department of Neurosurgery, Rennes University Hospital, rue Henri Le Guilloux, Rennes, France; 5 “MediCIS” laboratory (UMR 1099 LTSI), INSERM, University of Rennes 1, Avenue Léon Bernard, Rennes, France; 6 Department of Psychiatry, Rennes University Hospital, avenue du Général Leclerc, Rennes, France; 7 Department of Oncology, Eugene Marquis Center, Avenue de la Bataille Flandres-Dunkerque, Rennes, France; INSERM / CNRS, FRANCE

## Abstract

The mechanisms behind weight gain following deep brain stimulation (DBS) surgery seem to be multifactorial and suspected depending on the target, either the subthalamic nucleus (STN) or the globus pallidus internus (GPi). Decreased energy expenditure following motor improvement and behavioral and/or metabolic changes are possible explanations. Focusing on GPi target, our objective was to analyze correlations between changes in brain metabolism (measured with PET) and weight gain following GPi-DBS in patients with Parkinson’s disease (PD). Body mass index was calculated and brain activity prospectively measured using 2-deoxy-2[18F]fluoro-D-glucose PET four months before and four months after the start of GPi-DBS in 19 PD patients. Dopaminergic medication was included in the analysis to control for its possible influence on brain metabolism. Body mass index increased significantly by 0.66 ± 1.3 kg/m2 (*p* = 0.040). There were correlations between weight gain and changes in brain metabolism in premotor areas, including the left and right superior gyri (Brodmann area, BA 6), left superior gyrus (BA 8), the dorsolateral prefrontal cortex (right middle gyrus, BAs 9 and 46), and the left and right somatosensory association cortices (BA 7). However, we found no correlation between weight gain and metabolic changes in limbic and associative areas. Additionally, there was a trend toward a correlation between reduced dyskinesia and weight gain (*r* = 0.428, *p* = 0.067). These findings suggest that, unlike STN-DBS, motor improvement is the major contributing factor for weight gain following GPi-DBS PD, confirming the motor selectivity of this target.

## Introduction

Recent studies have shown that there is no single explanation for weight gain following deep brain stimulation (DBS) for Parkinson's disease (PD) [1,2]. Weight gain does not appear to be due simply to a reduction in motor symptoms, in turn inducing a decrease in energy expenditure [3–5]. Subthalamic nucleus (STN) DBS has been hypothesized to affect the central regulation of eating behavior and/or energy metabolism [4,6,7]. In this context, we investigated the relationship between changes in brain metabolism and changes in weight with STN DBS in patients with PD using positron emission tomography (PET) [8]. We found that weight gain correlated with metabolic changes in a distributed ventral associative-limbic network that encompassed the orbitofrontal, cingulate and temporal cortices. These structures are believed to be involved in the integration of sensory information with cognitive and affective representations of food, and the initiation of the behavioral responses needed to seek and obtain that food. By contrast, we found no correlation between weight change and motor areas. In an earlier study, we had observed that weight gain was only correlated with motor improvements (reduced dyskinesia) in the group of patients who underwent pallidal stimulation [9]. These results would suggest that, unlike weight gain following STN DBS, weight gain with pallidal DBS is mainly related to motor improvement, which then leads to a reduction in energy expenditure. To further explore this hypothesis, we prospectively analyzed correlations between changes in brain metabolism (using PET), and changes in body mass index (BMI) following DBS of the globus pallidus internalis (GPi) in patients with PD.

## Methods

### Subjects

The present study received approval from the local Ethical committee of the University Hospital of Rennes and was conducted in accordance with the Declaration of Helsinki and current French legislation (Huriet Act). After a complete description of the study, written informed consent was obtained from each patient. Nineteen patients (9 men, mean age at surgery: 61 ± 8 years) with idiopathic PD assigned to bilateral GPi DBS were included in our study. Table 1 summarizes the clinical characteristics of the patients before surgery. Prior to DBS, all the patients underwent neuropsychological and psychiatric assessments to rule out dementia and psychiatric disorders. DBS was indicated for disabling tremor, motor fluctuation and/or dyskinesia despite optimum drug treatment. A decrease of more than 50% in the Unified Parkinson’s Disease Rating Scale (UPDRS) Part III following an acute levodopa challenge was required for surgical eligibility. STN DBS was contraindicated for all patients, owing to cognitive impairment (Mattis Dementia Rating Scale, MDRS ≤ 130 or impaired executive functions) and/or dopa-resistant axial motor symptoms (dysarthria, freezing, falls) at baseline. Attribution to either STN or GPi DBS was supported by studies suggesting that the latter should be preferred in the case of mild cognitive impairment, as GPi DBS has fewer adverse effects on cognitive functions and behavior [10–12]. Each patient was assessed prospectively, before and after surgery, with all the motor, neuropsychological, psychiatric, weight and PET assessments being carried out within a one-week period. The mean ± *SD* intervals were 4 ± 3 months before implantation for the preoperative assessment and 4 ± 2 months after implantation for the postoperative assessment. The motor assessment was performed both on (“On drug”) and off (“Off drug”) dopaminergic medication, as well as “On DBS” and “Off DBS”. All patients were On drug (and On DBS postoperatively) for the PET scans.

**Table 1:** Clinical characteristics of the 19 patients before and after DBS surgery: mean ± SD scores.

	Preoperative assessment		Postoperative assessment			
	Off drug	On drug	Off drug / On DBS	Significant difference from Off drug preop. assessment	On drug / On DBS	Significant difference from On drug preop. assessment
**H&Y (/5)**	3.1 ± 1	1.9 ± 1	2.8 ± 1	ns	1.5 ± 1	ns
**S&E (/100%)**	54 ± 20	84 ± 13	64 ± 26	ns	89 ± 7	ns
**UPDRS-II (/52)**	22 ± 7	9 ± 6	18 ± 7	(p = 0.031)	8 ± 4	ns
**UPDRS-III (/108)**	41 ± 15	13 ± 5	25 ± 11	(p = 0.002)	12 ± 6	ns
**Total UPDRS-IV (/23)**		9 ± 3			4 ± 2	(p < 0.001)
**UPDRS-IV "Dysk" (/13)**		5 ± 3			1 ± 1	(p < 0.001)
**UPDRS-IV "Fluct" (/7)**		3 ± 1			2 ± 2	ns
**LEDD (mg)**		1415 ± 587			1372 ± 434	ns
**MDRS**		133 ± 8			132 ± 9	ns
**AES**		37 ± 6			38 ± 8	ns
**MADRS**		10 ± 6			10 ± 9	ns
**BMI (kg/m**^**2**^**)**		20.9 ± 3			21.7 ± 4	(p = 0.040)
**DEI (kcal/day)**		2634 ± 759			2564 ± 508	ns

H& Y = Hoehn and Yahr; S&E = Schwab and England; UPDRS = Unified Parkinson’s Disease Rating Scale; LEDD = L-dopa-equivalent daily dose; MDRS = Mattis Dementia Rating Scale; AES: Apathy Evaluation Scale; MADRS, Montgomery and Asberg Depression Rating Scale; BMI = body mass index; DEI = daily energy intake.

### Neurosurgery and stimulation settings

Surgery was performed under local anesthesia, using MRI determination of the target and intraoperative assessment of the clinical effects of stimulation. The correct position of the electrodes was checked postoperatively using a 3D CT brain scan. Quadripolar electrodes (3387; Medtronic, Minneapolis, MN, USA) were implanted bilaterally in all the patients. At four months after surgery, the selected contacts were located 23.5 ± 2.4 mm lateral to the anterior-posterior commissure (ACPC) line, 10.95 ± 1.86 mm posterior to the anterior commissure, and 0.2 ± 2.9 mm above the ACPC line. The stimulation parameters at that point were monopolar for 27 electrodes (voltage 2.6 ± 0.3 V; pulse width 76 ± 19 μs and frequency 131 ± 3 Hz) and bipolar for 11 electrodes (voltage 2.6 ± 0.5 V; pulse width 85 ± 23 μs and frequency 130 ± 0 Hz). Mean right and left stimulation voltages were similar (2.6 V ± 0.4 on both sides).

### Clinical assessment

All patients were assessed pre- and postoperatively according to the Core Assessment Program for Intracerebral Transplantation, and scored in the different stimulation and dopaminergic medication conditions. Motor function was assessed using the UPDRS and Levodopa-equivalent daily dose (LEDD), including the dose of dopaminergic agonists, was calculated according to Tomlinson et coll. [13]. Body weight was measured in the morning, after night fasting, on the same bathroom scales for all patients. BMI (kg/m^2^) and daily energy intake (kcal/day) were assessed prospectively for all patients before surgery, then at the time of the postoperative PET scan. A structured dietary questionnaire, assessing dietary habits and physical activity over a 7-day period, was administered to all patients by the same dietician. This dietician was especially instructed to detect any modification of eating behavior in the patients included in this study.

### PET imaging procedure

Patients were night fasted but had their usual dopaminergic medication for the PET scans. There was no statistical difference in fasting serum glucose levels at the time of the PET measurements, either before or after implantation. These were performed using a dedicated Discovery ST PET scanner (GEMS, Milwaukee, MN, USA) in 2D mode, with an axial field of view of 15.2 cm. A 222–296 MBq injection of 2-deoxy-2[18F]fluoro-D-glucose was administered intravenously in a resting state. A 20-minute 2D emission scan was performed 30 minutes post injection and after X-ray based attenuation correction. Patients were at rest during the imaging procedure. Following scatter, deadtime and random corrections, PET images were reconstructed by 2D filtered back-projection, providing 47 contiguous, transaxial 3.75-mm thick slices.

### PET image transformation

We used the same method as that described in our previous study [8,14]. The data were analyzed with statistical parametric mapping (SPM2; Wellcome Department of Cognitive Neurology, London, UK) implemented in MATLAB Version 7 (MathWorks Inc., Sherborn, MA, USA). Statistical parametric maps are spatially extended statistical processes used to characterize regionally specific effects in imaging data. SPM combines the general linear model (to create the statistical map) and the theory of Gaussian fields to make statistical inferences about regional effects. Images were first realigned and spatially normalized into standard stereotactic space (Talairach and Tournoux atlas). Affine transformation was performed to determine the 12 optimum parameters for registering the brain to the template. The subtle differences between the transformed image and the template were then removed using a nonlinear registration method. Finally, the spatially normalized images were smoothed using an isotropic 12-mm full width at half-maximum isotropic Gaussian kernel to compensate for interindividual anatomical variability and to render the imaging data more normally distributed.

### Data analysis

The SPM software established correlations between postoperative changes in the patients’ BMI scores and postoperative changes in their brain glucose metabolism. To identify which regions correlated significantly with increased BMI scores, the general linear “multisubject conditions and covariates” model was tested at each voxel with the BMI score as a covariate. LEDD was included in the SPM analysis as covariate, on the strength of reports in the literature underlining the potential confounding role of dopaminergic medication in regional metabolic changes and weight changes [3–5,15]. This yielded a regression coefficient that was then transformed into a *t* value. Two *t* tests were performed, one looking for positive correlations between increased BMI scores and increased voxel values, the other looking for negative correlations between increased BMI scores and decreased voxel values. *T* statistic SPMs were then calculated and thresholded at *p* < 0.001 (cluster-corrected) and k > 70 (expected number of voxels per cluster in SPM).

## Results

### Clinical assessment

**Fig 1:**
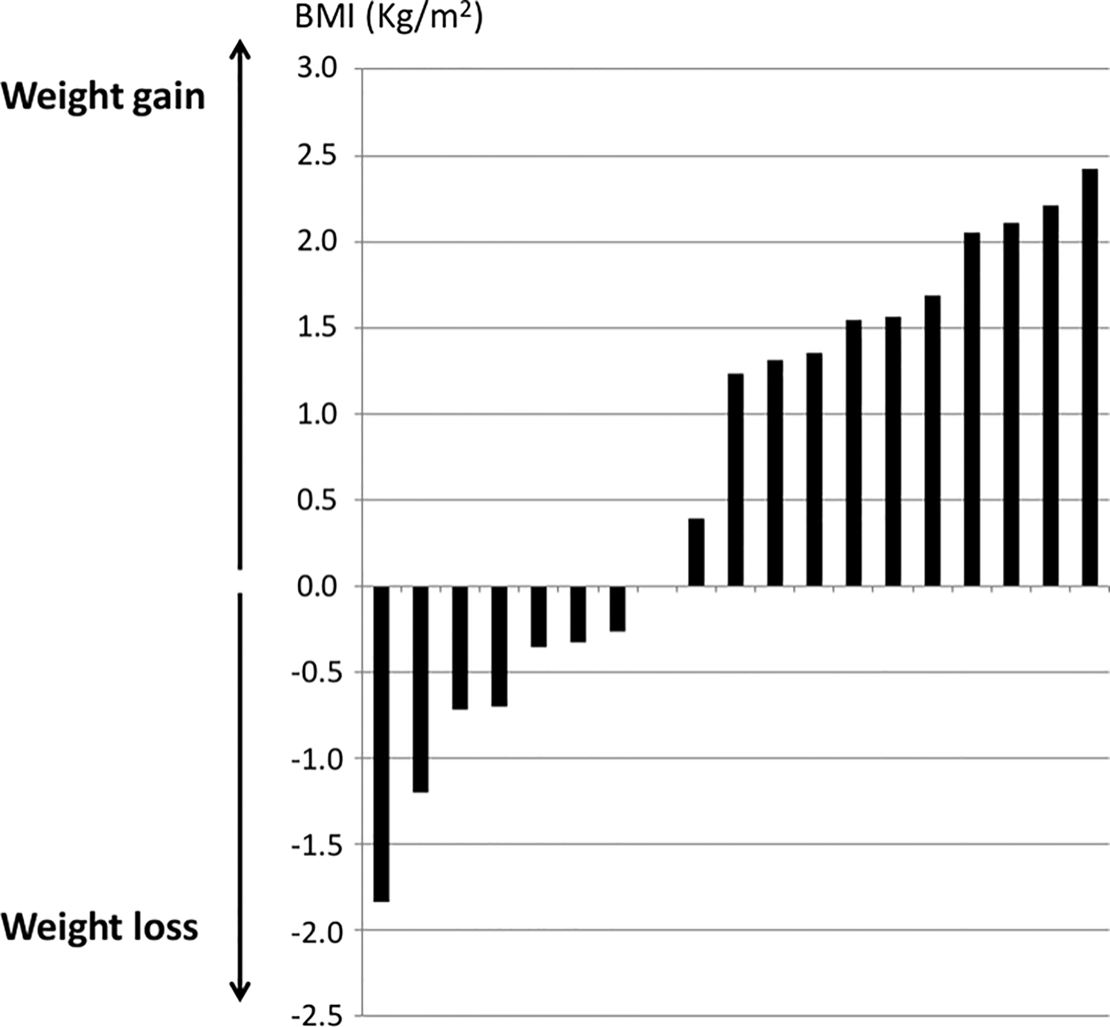
Distribution of changes in BMI across patients.

Motor improvements following DBS were highlighted by a significant decrease in the UPDRS-III score between the preoperative Off drug and postoperative Off drug/On DBS assessments, and significant decreases in the UPDRS-IV total score and dyskinesias subscore (Table 1 and S1 Table). There was no cognitive deterioration following DBS (MDRS = 133 ± 8 before and 132 ± 9 after DBS, *p* = 0.244). By Month 4, mean BMI had increased significantly by +0.66 ± 1.3 kg/m^2^ (*p* = 0.040). This corresponded to a mean increase of 1.7 ± 3.6 kg. BMI increased in 12 patients (63% of patients) and decreased in seven patients (37%) (Fig 1). Both groups did not differ statically for LEDD and UPDRS IV total score. The preoperative MDRS score was higher (138.7 ± 2.4 vs. 129. 6 ± 7.5, p = 0.007) as well as the preoperative Schwab and England (S&E) score (91.4 ± 10,7 vs. 80.0 ± 13.5, p = 0.048) in patients who lost weight. There was no other statistical difference between both groups, especially regarding the other scales of disease severity. The MDRS and the Apathy Evaluation Scale (AES) scores remained unchanged in both groups after surgery. The Montgomery and Asberg Depression Rating Scale (MADRS) score was not different between groups before surgery but decreased postoperatively in patients who lost weight (from 11 ± 7 to 6 ± 2, p = 0.028) while it remained unchanged in patients who gained weight (from 10 ± 6 to 10 ± 9, p = 0.266).

There was a higher but non-statically significant reduction in dyskinesias in patients who gained weight (-4.6 vs. -2.7). A gain in weight ≥ 5 kg (corresponding to a mean increase in the BMI of 2 points) occurred in four patients (21%). The mean change in BMI did not differ statistically between men (+0.6 ± 1.4 kg/m^2^) and women (+0.7 ± 1.3 kg/m^2^). Daily energy intake did not change statistically (-70 kcal/day, *p* = 0.609) and changes in BMI did not correlate with changes in daily energy intake (*r* = 0,036, *p* = 0,882). We found no change in dietary habits in these patients. Total dopaminergic medication remained unchanged (1415 ± 587 mg before and 1372 ± 434 mg after surgery, *p* = 0.758). This corresponded to a mean LEED/kg of 24.4 ± 12 mg/kg before surgery and of 22.7 ± 7 mg/kg after surgery (p = 0.47). Agonist medication was reduced from 542 ± 466 to 264 ± 222 mg (-51%, *p* = 0.013). The BMI increase did not correlate with changes in either the UPDRS II or III (On or Off drug/DBS) scores or subscores, UPDRS-IV Fluctuations subscore, UPDRS-IV total score or total agonist medication. However, there was a trend toward a correlation between an increase in BMI and a reduction in dyskinesias (Items 32–35 of the UPDRS) (*r* = 0.428, *p* = 0.067).

### PET results

**Fig 2:**
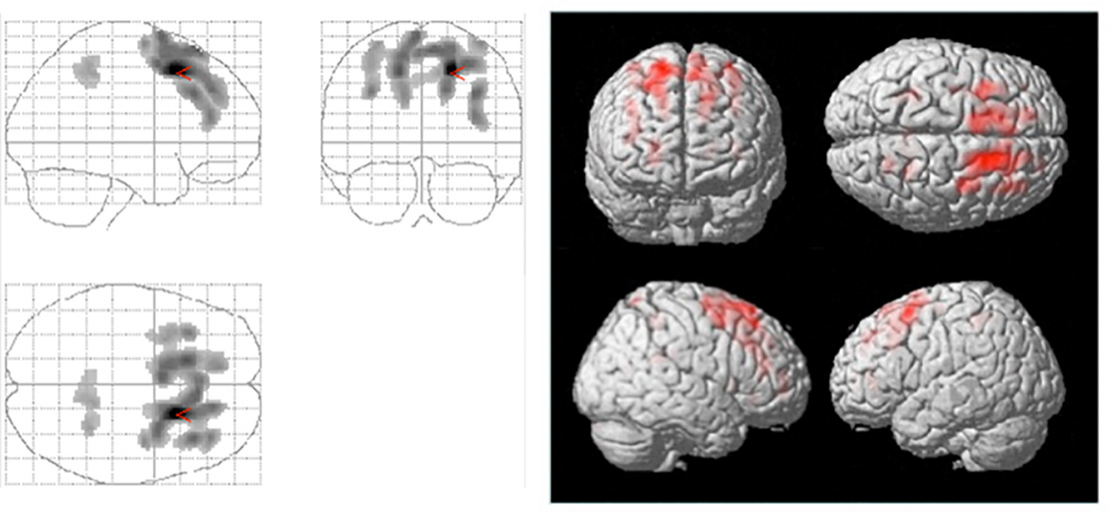
Positive correlations between increases in BMI and brain glucose metabolism assessed by PET (left), with corresponding 3D surface projections (right).

We found a number of clusters with positive correlations between increased BMI and increased metabolism (Table 2 and Fig 2). These correlations were observed in the frontal cortex, more specifically the left and right superior gyri (Brodmann area, BA 6), left superior gyrus (BA 8), right middle gyrus (BAs 9 and 46), and left and right parietal cortex (precuneus, BA 7). No negative correlation between changes in BMI and brain metabolism was observed.

**Table 2:** Summary of the analysis of correlations between increased brain glucose metabolism and increased BMI.

	Talairach coordinates				
Region	x	Y	z	z value	No. voxels
**Right frontal lobe, superior gyrus, BA 6**	20	16	48	5.34	4023
**Left frontal lobe, superior gyrus, BA 8**	-16	22	48	4.53	4023
**Left frontal lobe, superior gyrus, BA 6**	-4	6	72	4.39	4023
**Right frontal lobe, middle gyrus, BA 9**	38	26	34	4.34	476
**Right frontal lobe, middle gyrus, BA 46**	36	38	18	4.18	476
**Right parietal lobe, precuneus, BA 7**	12	-42	46	3.70	404
**Left parietal lobe, precuneus, BA 7**	-2	-46	42	3.29	404

Coordinates were based on the Talairach atlas and transformed by applying procedures developed by Matthew Brett (http://www.mrc-cbu.cam.ac.uk/Imaging). BA = Brodmann area. p < 0.001, multiple comparison corrected, at cluster level k > 70.

## Discussion

Data on weight gain following pallidotomy or GPi DBS are not abundant, and there are mixed reports on the extent of weight gain in patients undergoing GPi DBS. Several studies suggest that weight gain is lower after GPi surgery than after STN DBS [9,15–21], although other studies have failed to find any difference between the two targets [22,23]. As already observed in patients who underwent STN-DBS [8], some patients in the current study gained weight while others lost weight. This reminds the heterogeneity in weight changes observed in Parkinson’s disease, independently of DBS or following DBS [1].

In the present study, we assessed the pattern of brain metabolism associated with weight gain in Parkinsonian patients following GPi DBS. Of importance is to note that in our institution Pallidal DBS is an alternative to STN-DBS for patients with contraindications for subthalamic stimulation. This explains why the patients in the current study differ from the patients commonly selected for DBS, notably regarding higher disease severity and lower preoperative body weight as well as the absence of decrease in medication following surgery. We found that BMI changes correlated with changes in the metabolism of areas directly or indirectly involved in motor execution or programming on both sides, the dorsolateral prefrontal cortices (BAs 9, 46), premotor cortices (BAs 6, 8) and somatosensory association cortices (BA 7) but not of structures involved in associative or limbic functions. Further, there was no neuropsychological change that explained weight gain in these patients. The depression score decreased in patients who lost weight after surgery but not in patients who gained weight. This could suggest that mood improvement could play a role in weight loss in some patients. However, we did not observe any relation between psychological changes and weight gain following pallidal DBS. Dopaminergic medication was included in the analysis, in order to control for its possible influence on brain metabolism and weight gain. In addition to the avoidance of confounding factors such as the introduction of dietary management, the short follow-up duration of this study strengthens the argument in favor of a specific causative effect of surgery on weight gain and it is unlikely than other factors have contributed to weight changes. Though the duration of the study and the sample of patients might somewhat limit the interpretation of the results, this study supports our hypothesis that weight gain following pallidal DBS in PD patients is related more to motor improvement than to either behavioral or metabolic factors. Furthermore, it suggests that although weight gain is commonly observed whatever the surgical target, the main mechanism underlying weight gain probably differs according to the nature of that target.

In a previous study, we had observed that changes in BMI were correlated with reduced dyskinesia in PD patients undergoing pallidal DBS [9]. This suggested that it was diminished energy expenditure following motor improvement that contributed to weight gain in these patients. In the current study, involving a different patient sample, we observed a trend toward a correlation between the reduction in dyskinesias and weight gain. Using PET imaging in these patients, we observed a correlation between changes in weight and changes in brain metabolism in dorsolateral prefrontal, premotor and sensorimotor areas, all these areas being involved in motor execution or programming. Taken together, these two studies suggest that motor improvement is a major factor for weight gain in patients with PD undergoing pallidal DBS. This is in accordance with a recent study in patients with dystonia that confirms that reduction of motor symptoms following GPi DBS results in weight gain [24]. Regarding the precise nature of the influence of motor improvement on weight gain in PD patients, Ondo et al. reported that weight gain following unilateral pallidotomy correlated with improvements in the Off and On drug UPDRS-III scores [16]. Another study found a correlation between weight gain and improvements in UPDRS-III scores and dyskinesias, although the surgical procedure (nine unilateral pallidotomy, nine bilateral pallidal DBS, nine bilateral STN DBS) was too heterogeneous to allow any firm conclusions to be drawn [15]. The results from Lang et al. are similarly difficult to interpret, as weight gain occurred in association with various neuropsychological changes [18]. In other studies, no correlation was found between motor improvement and weight gain in GPi surgery [21–23]. The same inconsistency prevails in studies of weight gain in STN DBS patients, and the possible impact of decreased energy expenditure resulting from motor improvement is still being debated. As Kistner et al. noted, a reduction in energy expenditure could have a number of explanations, including improvements in rigidity, tremor, dyskinesia, OFF-period dystonia, and nocturnal hyperactivity [2]. There have been several reports of reduced energy expenditure after STN DBS for PD [4,5], and some studies have reported less frequent or less severe dyskinesias in relation to weight gain [3,9,15,23].

Motor improvement is not considered to be a sufficient explanation for weight gain with STN DBS, and several additional ones have been put forward. Although no firm conclusion has yet been drawn, there is a conceptual view than weight gain following STN DBS for PD is due to a combination of factors [1,2]. Unfortunately, information on such potential factors is lacking in the context of GPi DBS. To our knowledge, there have not been any studies of metabolism in pallidal DBS as there have in STN DBS. However, some factors hypothesized to contribute to weight gain after STN DBS are unlikely to concern GPi DBS-treated patients. First, unlike STN DBS [25–27], eating disorders have not been described in GPi DBS-treated patients, although there have not been any studies specifically dealing with food habits in these patients. We did not find a relation between changes in DEI and weight gain that would have support this hypothesis, in the current or in our previous study [9]. In addition, though not specifically evaluated with standardized scales, there was no report of any change in dietary habits in these patients. The influence of dopamine withdrawal after surgery on eating disorders in the context of STN DBS has recently been reviewed [2]. However, the postoperative reduction in dopaminergic medication is moderate at most, in the case of pallidal surgery, and so-called *hypo-dopaminergic snacking* is unlikely to occur in GPi DBS-treated patients. Furthermore, dopaminergic agonists are known to contribute to weight gain in PD but as there were reduced postoperatively in our patients, this medication cannot be considered as a confounding factor in weight gain. Second, it has been suggested that STN stimulation has a direct effect on the hypothalamus through current diffusion [28]. Like other researchers, we think this unlikely [2]. It seems more plausible that current diffusion to the associative-limbic compartment of the STN induces changes in homeostatic hypothalamic regulation through the limbic circuitry of the basal ganglia. In GPi DBS, a similar effect is unlikely to occur, owing to the size of the pallidum, even assuming that there is current diffusion to the ventral pallidum. Both the ventral striatum and the ventral pallidum have been implicated in eating behavior through their projections to the lateral hypothalamic area [29]. Changes in eating behavior have been observed when the neural activity of the shell subregion of the nucleus accumbens or ventral pallidum is manipulated [30]. These effects are assumed to be mediated through the lateral hypothalamic area. However, there is no evidence that GPi DBS influences either of these structures.

In conclusion, although the mechanisms behind weight gain following surgery for PD are not yet fully understood, many authors consider them to be multifactorial. This would explain the inconsistent results in the literature, be it for weight gain in DBS or for the extent of weight gain in GPi DBS relative to STN DBS. Though we did not perform a direct comparison of patients undergoing STN-DBS or pallidal DBS, both the current study and the similar study we conducted in PD patients undergoing STN DBS [8] suggest that the main mechanism behind weight gain differs according to the target. In the case of STN DBS, we found that weight gain correlated with changes in brain metabolism in associative and limbic areas, and suggested that motor improvement was therefore not the main factor leading to weight gain in STN DBS-treated patients [8]. Without ruling out a role for motor improvement, we considered weight gain to be linked mainly to changes or nonadaptation in the regulation of eating and/or metabolism. By contrast, the current study suggests that motor improvement (and the presumably attendant reduction in energy expenditure) is the main factor for weight gain in the case of pallidal DBS. There is a rationale for such a difference. The STN is a small structure, and current diffusion to associative and limbic compartments is plausible. By contrast, owing to the size of the GPi, DBS of the sensorimotor compartment of the GPi is unlikely to affect other functions, confirming the motor selectivity of this target. At last, we suggest that the mechanisms behind weight gain following DBS differ depending on the target being implanted.

## Supporting Information

S1 Table Individual clinical and demographic characteristics of the patients before and after GPi surgery. (XLSX)
